# Programmable Macrophage Mimics for Inflammatory Meniscus Regeneration via Nanotherapy

**DOI:** 10.34133/research.1056

**Published:** 2026-01-12

**Authors:** Xujie Lu, Zheng Ci, Bohui Li, Yajie Wang, Di Wang, Xiang Zhang, Yingying Huo, Xiansong Wang, Guangdong Zhou, Yujie Hua

**Affiliations:** ^1^ Department of Plastic and Reconstructive Surgery, Shanghai Ninth People’s Hospital, Shanghai Key Laboratory of Tissue Engineering, Shanghai Jiao Tong University School of Medicine, Shanghai 200001, PR China.; ^2^ Plastic Surgery Institute, Shandong Provincial Key Laboratory for Tissue Regeneration and Repair & Reconstruction (Under Preparation), Shandong Second Medical University, Weifang, Shandong 261053, PR China.; ^3^ National Tissue Engineering Center of China, Shanghai 200241, PR China.

## Abstract

Meniscal injuries are common in the knee joint. Minor meniscal injuries usually respond well to conservative treatment, while severe cases often require complete meniscal replacement. Meniscal injuries cause inflammatory responses that importantly hinder meniscal tissue regeneration. Despite ongoing advances in research, considerable breakthroughs in meniscal regeneration remain out of reach. This study introduces programmable macrophage mimics (PMMs), which enable sequential regulation from anti-inflammatory responses to meniscal fibrocartilage regeneration. PMMs were prepared by encapsulating the transforming growth factor-β3 and insulin-like growth factor-1 growth factors within mesoporous silica nanoparticles modified with branched polyethyleneimine via disulfide bonding. This design allows the initial adsorption of proinflammatory cytokines followed by the controlled release of growth factors that promote adipose-derived stem cell (ADSC) differentiation into fibrochondrocytes. The PMMs were integrated into meniscus-specific acellular matrix hydrogels (mGC), which provided suitable mechanical properties critical for effective regeneration. In rabbit osteoarthritis models, ADSC-loaded PMMs@mGC hydrogels showed marked fibrocartilage regeneration. Additionally, the team developed an advanced biofabrication approach that combines a 3-dimensionally printed polycaprolactone framework designed for total meniscus replacement. This research suggests that PMMs act as a bifunctional “core–shell” nano-delivery system, offering a promising therapeutic strategy for managing inflammatory meniscal conditions.

## Introduction

The meniscus is a fibrocartilaginous tissue and organ in the human knee joint that serves critical functions, including load transmission, shock absorption, joint stability, and lubrication. Meniscal injuries are among the most common knee injuries, typically caused by acute trauma or age-related degeneration [1–3]. Minor meniscal injuries are usually treated with in situ arthroscopic procedures or conservative methods, whereas larger or more severe injuries often necessitate total meniscus replacement. Recent advances in materials science and manufacturing techniques have enabled transformative tissue-engineering strategies for meniscal therapy [4,5]. Several stem cell types, including synovium-derived mesenchymal stem cells, bone-marrow-derived mesenchymal stem cells, and adipose-derived stem cells (ADSCs), have been investigated as candidate seed cells for meniscal regeneration and repair. Notably, ADSCs are clinically promising because of their ease of harvest, high inducibility, innate anti-inflammatory properties, and potential to promote fibrocartilage regeneration [6–8]. Our group has developed a series of decellularized matrix scaffolds for auricular, nasal, tracheal, and articular cartilage repair using 3-dimensional (3D) bioprinting techniques, successfully repairing meniscus defects and restoring physiological function [9–12]. However, current tissue-engineering strategies for meniscus defect repair commonly rely on a favorable regenerative microenvironment. Pathological conditions such as osteoarthritis (OA) [13–16], the most prevalent joint disorder, often create inflammatory environments that severely hinder meniscus regeneration [17–21]. Moreover, meniscal injury exacerbates the local inflammatory milieu, further impeding tissue healing and inevitably accelerating OA progression. Therefore, there is an urgent need to establish a cartilaginous immune microenvironment that first mitigates early-stage inflammation after meniscal injury and then sequentially promotes later-stage fibrocartilage regeneration [22–25].

Currently, targeted regulation using small-molecule drug injections is commonly employed to treat inflammatory conditions in sports medicine [26,27]. Most of these drugs exhibit broad-spectrum anti-inflammatory effects and inevitably cause varying degrees of side effects by activating nonspecific signaling pathways. Polyethyleneimine is a highly cationic polymer. It is widely used to modulate inflammation by adsorbing and removing negatively charged proinflammatory cytokines, including tumor necrosis factor-α (TNF-α), interleukin-1β (IL-1β), and interleukin-6 (IL-6), via electrostatic interactions [28–31]. Notably, modifying polyethyleneimine into its branched form (branched polyethyleneimine [BPEI]) has been shown to improve cytocompatibility and enhance in vivo metabolic cycling. Regarding fibrocartilage regeneration, growth factors such as transforming growth factor-β3 (TGFβ3) and insulin-like growth factor-1 (IGF1) are key inducers that drive stem cell differentiation toward a meniscus-specific fibrochondrocyte phenotype [32–34]. However, current drug delivery systems often fail to achieve controlled release of these growth factors, resulting in unsatisfactory long-term induction of differentiation and regenerative repair outcomes [35–37]. Recently, the artificial cell-based design concept has drawn significant attention as a promising direction for next-generation innovative, responsive materials [38–41]. In essence, whole biological cell mimics with cell-like structures can replicate the essential characteristics and biological functions of various living cells. In contrast, traditional engineered materials typically mimic only certain physical or structural traits [42–46]. To date, there have been few reports on creating cell mimics with immune-cell-related functions via nanotechnology. Recent studies have demonstrated that nanotherapeutic strategies incorporating immune-cell-mimicking designs can effectively suppress inflammatory responses and synergistically promote tissue regeneration [47–49]. Macrophages, which are key immune cells that regulate early-stage inflammation, play a central role in clearing proinflammatory cytokines. Accordingly, cell mimics with macrophage-like functions may offer therapeutic benefit for the treatment of inflammatory meniscal injury [50–52].

**Fig. 1.**
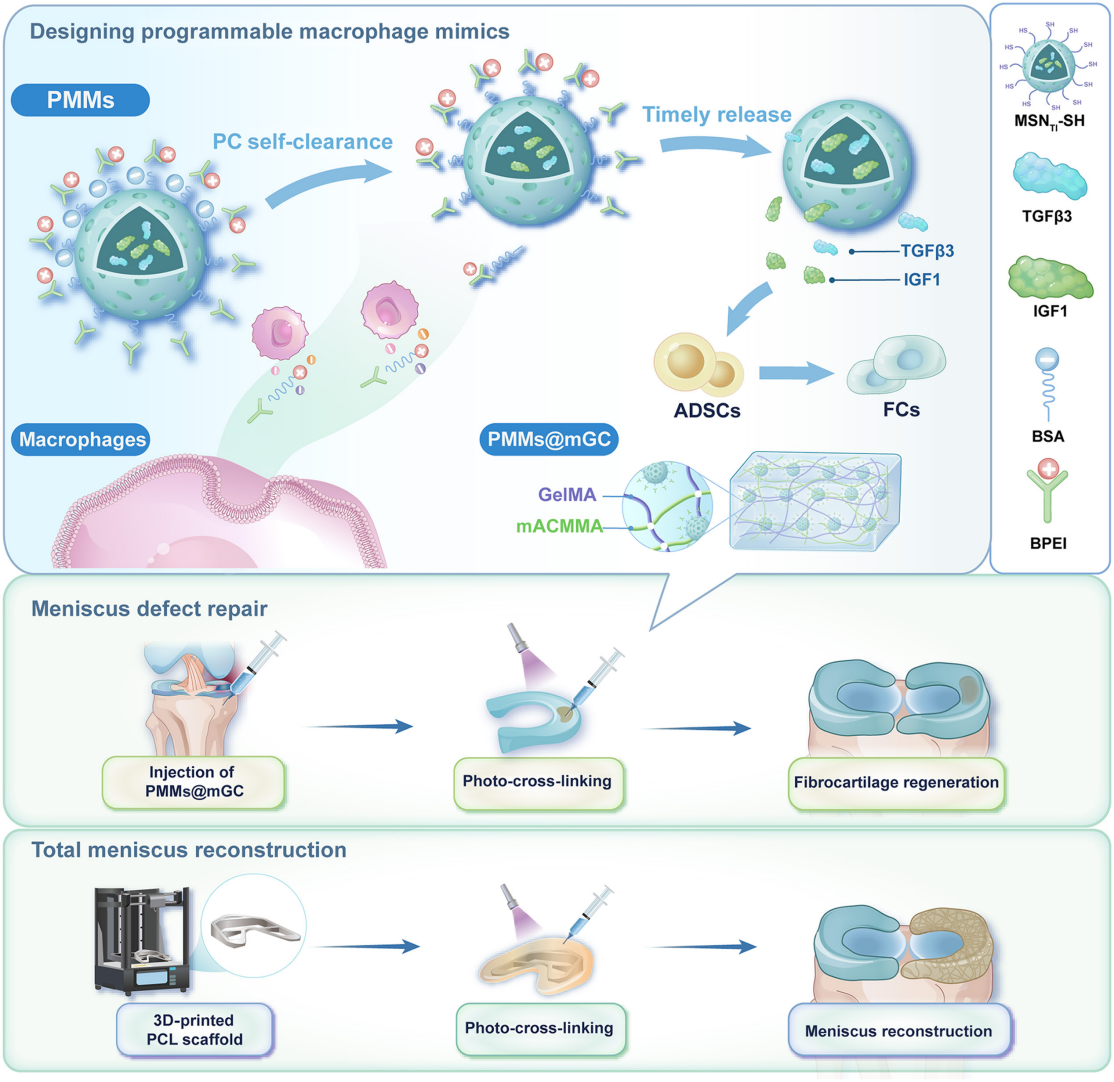
Schematic illustration of PMMs enabling cascade regulation from anti-inflammation to meniscus fibrocartilage regeneration in rabbit models, including both meniscus defect repair and total meniscus reconstruction. PMMs, programmable macrophage mimics; ADSCs, adipose-derived stem cells; FCs, fibrochondrocytes; PCs, proinflammatory cytokines; BSA, bovine serum albumin; BPEI, branched polyethylenimine; MSN_TI_-SH, transforming growth factor-β3 (TGFβ3)/insulin-like growth factor-1 (IGF1)-loaded thiol-modified mesoporous silica nanoparticles; GelMA, methacrylated gelatin; mACMMA, methacrylated meniscus cartilage-derived acellular matrix; 3D, 3-dimensional; PCL, polycaprolactone.

Here, we propose an emerging nanotherapeutic concept of programmable macrophage mimics (PMMs) that enable cascade regulation from anti-inflammation to meniscal fibrocartilage regeneration (Fig. 1). In this study, PMMs were constructed by encapsulating the fibrochondrogenic growth factors TGFβ3 and IGF1 within mesoporous silica nanoparticles (MSNs) and further modifying the outer layers with BPEI via disulfide bonding. The reversible grafting of BPEI allowed for responsive detachment in the inflammatory microenvironment, facilitating early-stage adsorption and clearance of proinflammatory cytokines. Subsequently, the TGFβ3/IGF1 composite growth factors were released promptly during the later regenerative period, inducing ADSC differentiation into fibrochondrocytes and promoting meniscus fibrocartilage regeneration. PMMs were further incorporated into meniscus-specific acellular matrix (mGC) hydrogels, providing suitable rheological and compressive properties for meniscus repair. In a rabbit OA model, ADSC-loaded PMMs@mGC hydrogels were rapidly cross-linked in situ to fill meniscus defects, resulting in satisfactory fibrocartilage regeneration. Additionally, a biofabrication strategy combining a crescent-shaped 3D-printed polycaprolactone (PCL) framework with ADSC-loaded PMMs@mGC hydrogels successfully achieved total meniscus replacement. Overall, the programmable macrophage effectively mimics early-stage proinflammatory cytokines following meniscal injury and subsequently promotes fibrocartilage regeneration, offering a promising strategy for comprehensive inflammatory meniscus therapy.

## Results

### Designing the PMMs

**Fig. 2.**
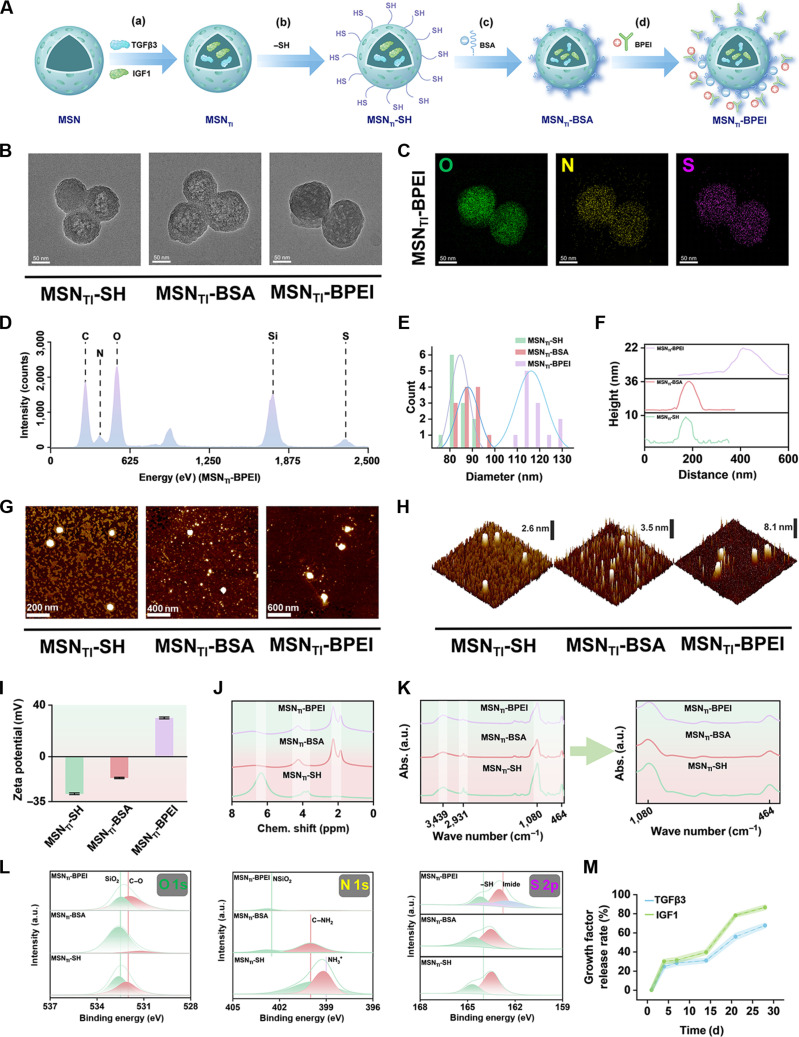
Preparation and characterization of PMMs. (A) Schematic illustration of the PMM preparation processes: (a) TGFβ3/IGF1 encapsulation, (b) thiol modification, (c) reversible BSA grafting, and (d) BPEI adsorption. (B) Representative transmission electron microscopy images of MSN_TI_-SH, MSN_TI_-BSA, and MSN_TI_-BPEI nanoparticles. (C) Representative energy-dispersive spectroscopy showing O, N, and S and (D) corresponding elemental percentages of MSN_TI_-BPEI nanoparticles. (E) Particle size distribution of MSN_TI_-SH, MSN_TI_-BSA, and MSN_TI_-BPEI nanoparticles. (F) Representative atomic force microscopy images of MSN_TI_-SH, MSN_TI_-BSA, and MSN_TI_-BPEI nanoparticles. (G) Surface thickness spectra and (H) corresponding morphological curves of MSN_TI_-SH, MSN_TI_-BSA, and MSN_TI_-BPEI nanoparticles. (I) Zeta potential variations, (J) solid-state nuclear magnetic resonance (NMR) spectra, (K) Fourier transform infrared spectroscopy, and (L) x-ray photoelectron spectroscopy of MSN_TI_-SH, MSN_TI_-BSA, and MSN_TI_-BPEI nanoparticles. X-ray photoelectron spectroscopy analysis includes O 1s, N 1s, and S 2p spectra. (M) Release profiles of TGFβ3 and IGF1 growth factors encapsulated in MSN_TI_-BPEI nanoparticles. MSN, mesoporous silica nanoparticles; MSN_TI_-SH, TGFβ3/IGF1-loaded thiol-modified MSN; MSN_TI_-BSA, TGFβ3/IGF1-loaded BSA-modified MSN; MSN_TI_-BPEI, TGFβ3/IGF1-loaded PEI-modified MSN; O, oxygen; N, nitrogen; S, sulfur.

Inspired by artificial cell-mimicking materials, we envisioned that it is essential to sequentially eliminate proinflammatory factors and induce fibrocartilage differentiation for effective regenerative therapy of the meniscus. To achieve this, we developed a cell-mimicking nanotherapeutic strategy (PMMs). We created PMMs that enable sequential regulation, beginning with inflammation suppression and culminating in meniscal fibrocartilage regeneration. As shown in Fig. 2A, PMMs were fabricated by encapsulating fibrocartilage-specific growth factors, TGFβ3 and IGF1, into MSN cores via ultrasonic treatment. Thiol modification of the MSN shell was then performed by stirring with mercaptopropyltrimethoxysilane, yielding MSN_TI_-SH. Subsequently, bovine serum albumin (BSA), containing free thiol groups, was reversibly grafted onto the MSNs via disulfide bonding to produce MSN_TI_-BSA. This was followed by the incorporation of the highly cationic polymer BPEI, resulting in a “core–shell” nanocarrier of MSN_TI_-BPEI. The reversible modification with BPEI was designed to adsorb and eliminate negatively charged inflammatory factors in the early period. Once this was achieved, the encapsulated TGFβ3/IGF1 growth factors were promptly released to promote fibrocartilage differentiation of ADSCs.

Furthermore, morphological features were first characterized using transmission electron microscopy (TEM) and atomic force microscopy. As shown in Fig. 2B, TEM images revealed that MSN_TI_-SH nanoparticles were generally spherical, indicating a well-developed porous structure suitable for encapsulating growth factors. Similarly, MSN_TI_-BSA and MSN_TI_-BPEI nanoparticles maintained a spherical morphology with uniform dispersibility, regardless of the outer modifications to the mesoporous silica surface. Energy-dispersive x-ray spectroscopy mapping and elemental intensity profiles confirmed that MSN_TI_-BPEI nanoparticles primarily consisted of the O, N, and S elements, in contrast to MSN_TI_-SH and MSN_TI_-BSA nanoparticles, which indirectly indicates successful BPEI modification through disulfide bonding (Fig. 2C and D and Fig. S1). For quantitative particle size analysis, MSN_TI_-BPEI nanoparticles exhibited the largest average diameter, approximately 118 nm, while MSN_TI_-SH and MSN_TI_-BSA nanoparticles measured around 86 and 89 nm, respectively (Fig. 2E). The increased size of MSN_TI_-BPEI nanoparticles was primarily attributed to the adsorption of the BPEI outer layer. Additionally, atomic force microscopic images validated the representative morphology and particle size findings for each group (Fig. 2G and H). Surface thickness measurements further confirmed a notable increase in MSN_TI_-BPEI nanoparticles (approximately 8.1 nm) following BPEI adsorption, compared with MSN_TI_-SH (approximately 3.5 nm) and MSN_TI_-BSA nanoparticles (approximately 2.6 nm) (Fig. 2F). Beyond morphological characterization, we assessed the nanoparticles’ surface potential, chemical composition, and growth factor release. Zeta potential analysis showed that MSN_TI_-SH and MSN_TI_-BSA nanoparticles were negatively charged. In contrast, MSN_TI_-BPEI nanoparticles became strongly positive following BPEI modification (Fig. 2I). This charge shift may facilitate the removal of negatively charged inflammatory factors. As shown in Fig. 2J, ^1^H nuclear magnetic resonance spectra confirmed the structural features of the 3 nanoparticle types. The peak at approximately 2 ppm corresponded to grafted BSA and protein amide bonds, while the peak near 6 ppm corresponded to silane sulfide bonds formed by thiol–silicon interactions. Fourier transform infrared spectroscopy further characterized the microspheres. As shown in Fig. 2K, MSN_TI_-SH nanoparticles displayed a peak at 1,080 cm^−1^, confirming thiol modification. MSN_TI_-BSA and MSN_TI_-BPEI nanoparticles showed reduced thiol peaks, likely due to surface coatings masking siloxane signals, thereby supporting successful BPEI attachment. The increased peak at 3,439 cm^−1^ in MSN_TI_-BPEI nanoparticles was attributed to BPEI amino groups. X-ray photoelectron spectroscopy profiles further confirmed surface composition (Fig. 2L and Fig. S2). MSN_TI_-BPEI nanoparticles exhibited a shift toward the C–O region, likely due to BPEI adsorption. Strong C–NH_2_ peaks were observed in both MSN_TI_-BSA and MSN_TI_-BPEI nanoparticles, but not in MSN_TI_-SH nanoparticles. S 2p doublets corresponding to –SH were present in MSN_TI_-SH and MSN_TI_-BSA nanoparticles but were attenuated in MSN_TI_-BPEI nanoparticles, consistent with surface coverage by BPEI. The 28-d release profiles of TGFβ3 and IGF1 from MSN_TI_-BPEI nanoparticles are shown in Fig. 2M. Approximately 28% of the growth factors were rapidly released during days 0 to 4, likely due to surface-adsorbed or incompletely encapsulated molecules. A stable release stage followed from days 5 to 14, attributed to BPEI encapsulation. From days 15 to 22, delayed release was observed as the BSA–BPEI interface was cleaved, followed by gradual release through day 28. These results indicate that MSN_TI_-BPEI nanoparticles efficiently scavenge negatively charged inflammatory factors and enable the sustained release of growth factors, supporting their role in constructing macrophage mimics for cascade regulation and fibrocartilage repair.

### Preparation and characterization of meniscus-specific hydrogels

**Fig. 3.**
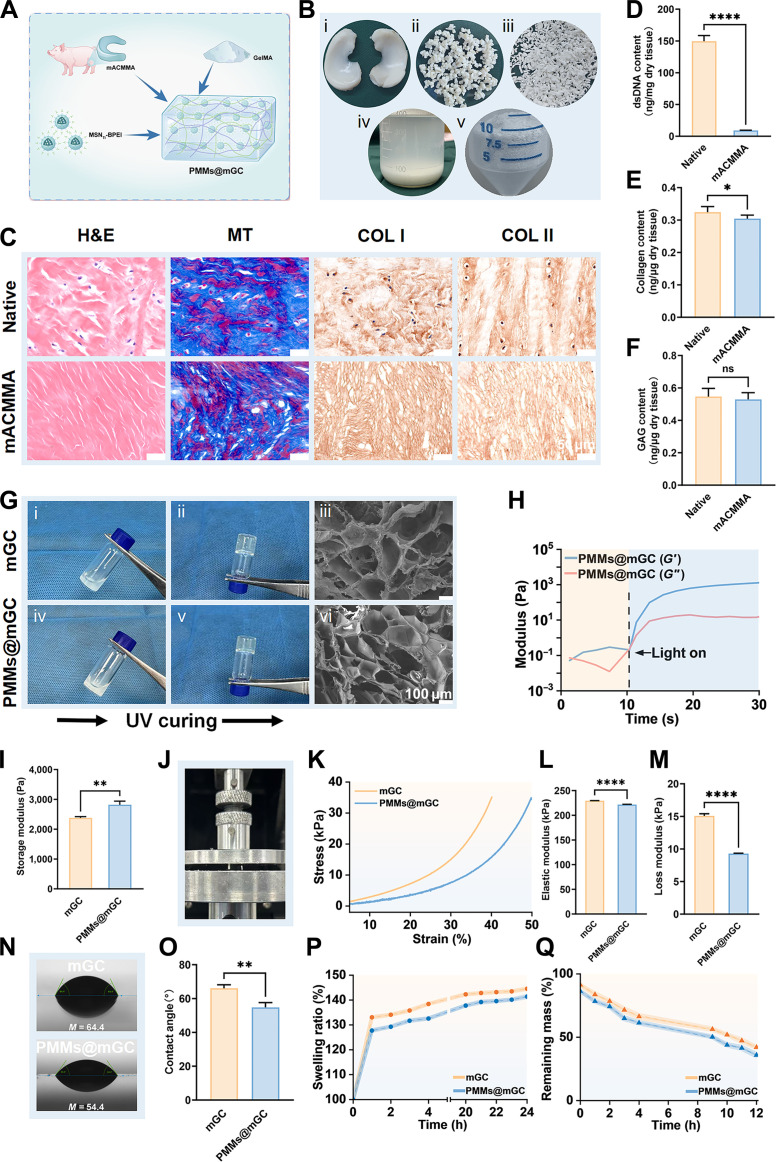
Preparation and characterization of meniscus-specific hydrogels. (A) Schematic illustration of the main components of the meniscus-specific hydrogels PMMs@mGC, including GelMA, mACMMA, and PMMs. (B) The preparation process of mACMMA, including (i) acquisition of porcine meniscus, (ii) decellularization, (iii) solubilization treatment, (iv) pepsin hydrolysis and methacrylate modification, and (v) lyophilization and milling into powder. (C) Histological and immunohistochemical analyses of native and acellular meniscus, including hematoxylin and eosin (H&E), Masson’s trichrome (MT), type I collagen (COL I), and type II collagen (COL II) staining. Quantitative statistics of (D) double-stranded DNA (dsDNA), (E) collagen, and (F) glycosaminoglycan (GAG) contents in meniscus tissue before and after decellularization. (G) Sol-to-gel transition of (i to iii) mGC and (iv to vi) PMMs@mGC hydrogels before and after photopolymerization (365-nm light-emitting diode [LED], 20 mW/cm^2^), along with scanning electron microscopy images of the corresponding lyophilized hydrogel scaffolds. (H) Rheological curves of PMMs@mGC hydrogels upon light irradiation (365-nm LED, 20 mW/cm^2^). (I) Statistical comparison of storage modulus between mGC and PMMs@mGC hydrogels. (J) Photograph of PMMs@mGC hydrogel under approximately 50% compressive strain, showing no breakage. (K) Representative compressive stress–strain curves of mGC and PMMs@mGC hydrogels. Statistical data of (L) elastic modulus and (M) compressive strength between mGC and PMMs@mGC hydrogels. Photographs of (N) water contact angle tests and (O) the corresponding contact angle measurements between mGC and PMMs@mGC hydrogels. Line graphs illustrating the (P) swelling ratio and (Q) degradation rate of mGC and PMMs@mGC hydrogels. mGC, 8% GelMA/1% mACMMA hydrogels; PMMs@mGC, 1% MSN_TI_-BPEI@8% GelMA/1% mACMMA hydrogels (w/v, weight/volume); UV, ultraviolet. Data are expressed as mean ± SD, *n* = 3. Statistical significance among various groups was determined by 1-way/2-way analysis of variance (ANOVA) and a *t* test.*P < 0.05, **P < 0.01, and ****P < 0.0001; ns, no significance.

Three-dimensional hydrogel-based cell culture supports stem cell differentiation and tissue regeneration [53,54]. In this study, PMMs were carefully designed to provide essential chemical cues for meniscal fibrocartilage regeneration, while hydrogel scaffolds were used to establish a 3D cell culture environment. To better mimic the meniscal fibrocartilage microenvironment, we selected porcine meniscus tissue to prepare a methacrylated meniscus cartilage-derived acellular matrix (mACMMA). The mACMMA polymer was combined with methacrylated gelatin (GelMA) to form mGC, with the addition of PMMs referred to as PMMs@mGC (Fig. 3A). The preparation of mACMMA involved these main steps: decellularization, enzymatic digestion, methacrylation, lyophilization, and milling into powder as depicted in Fig. 3B. Histological and immunohistochemical analyses of native and acellular meniscal tissue, which included hematoxylin and eosin (H&E) staining, Masson’s trichrome (MT) staining, and immunostaining for type I and type II collagen (COL I and COL II), confirmed successful decellularization. The acellular group showed no nuclear staining (Fig. 3C). Importantly, most collagen and fibrous components were reasonably preserved after decellularization, as evidenced by the positive staining for COL I and COL II proteins. Quantitative analyses were also conducted before and after decellularization to assess double-stranded DNA (dsDNA) levels, total collagen content, and glycosaminoglycan (GAG) content, as shown in Fig. 3D to F. The decellularized mACMMA retained most GAGs and collagen components while effectively removing dsDNA, thereby creating a favorable microenvironment for meniscal fibrocartilage repair.

Furthermore, the hybrid photo-cross-linkable hydrogels were characterized for their rheological, mechanical, and other physicochemical properties. As shown in Fig. 3G, both mGC and PMMs@mGC hydrogels cross-linked rapidly upon light irradiation (365-nm light-emitting diode [LED], 20 mW/cm^2^). Scanning electron microscope images displayed the corresponding lyophilized morphology of the hydrogel scaffolds. Figure 3H shows that the storage modulus (*G*′) quickly exceeded the loss modulus (*G*″) upon irradiation, confirming fast gelation and the formation of a stable hydrogel network. Comparative analysis of the storage modulus revealed that PMMs@mGC hydrogels reached 2,817 ± 122 Pa, higher than that of mGC hydrogels at 2,379 ± 41 Pa (Fig. 3I). This nano-enhancement of shear modulus was mainly attributed to the incorporation of high-modulus nanoparticles into the low-modulus hydrogel matrix. Stress–strain compressive testing demonstrated the elastic nature of both mGC and PMMs@mGC hydrogels (Fig. 3J to M). While the compressive modulus and strength of PMMs@mGC hydrogels were slightly lower than those of mGC hydrogels, they still met the basic mechanical requirements for meniscal fibrocartilage regeneration. Water contact angle measurements indicated that both hydrogel types had contact angles below 90°, suggesting moderate hydrophilicity conducive to cell adhesion (Fig. 3N and O). Additionally, no significant differences were observed in swelling ratio or degradation rate between mGC and PMMs@mGC hydrogels, regardless of PMM incorporation (Fig. 3P and Q). Notably, PMMs@mGC hydrogels degraded to approximately 30% under collagenase digestion, demonstrating a satisfactory biodegradability profile for tissue-engineered scaffolds. Overall, these findings indicate that our hydrogel scaffolds support rapid photopolymerization-mediated gelation and exhibit suitable rheological and compressive properties for meniscal fibrocartilage regeneration.

### Evaluation of inflammatory factor self-clearance and fibrocartilage induction

**Fig. 4.**
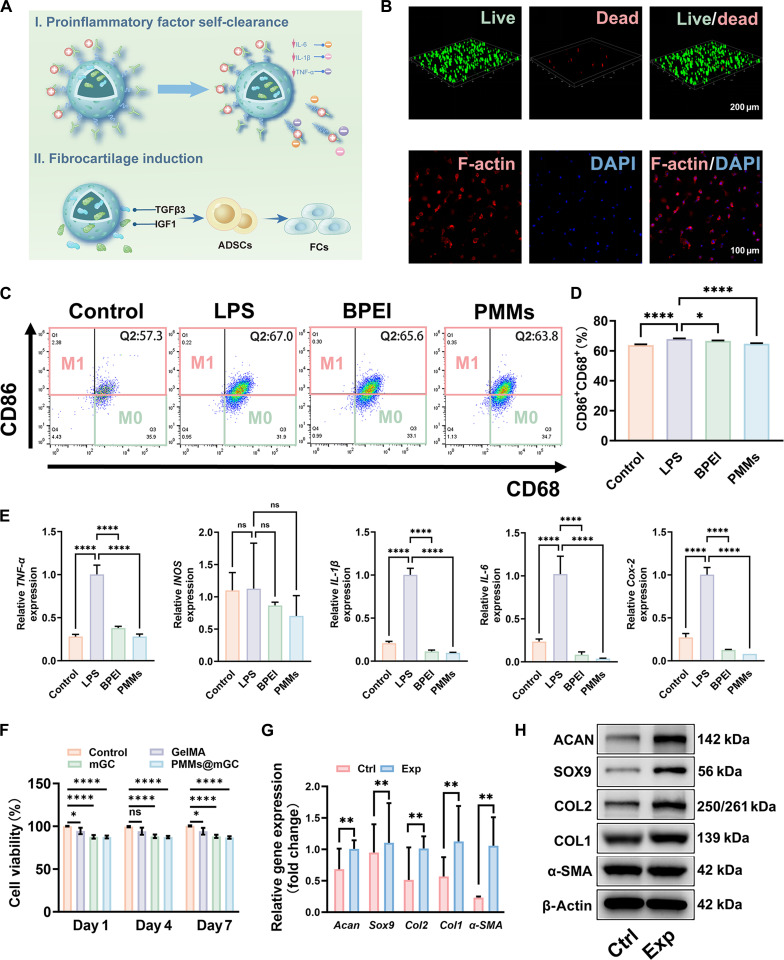
Biological evaluation of proinflammatory factor self-clearance and fibrocartilage induction. (A) Schematic illustration of the “immuno-regenerative” cascade regulation strategy using macrophage-mimicking nanomaterials, involving 2 stages: (I) proinflammatory factor self-clearance and (II) fibrocartilage induction. (B) Live/dead staining and F-actin staining of ADSCs cultured in PMMs@mGC hydrogels for 7 d. (C) Flow cytometry analysis of proinflammatory factor self-clearance capacity following lipopolysaccharide (LPS)-induced M1 macrophage polarization, treated with BPEI or MSN_TI_-BPEI. (D) Histogram showing the percentage of M1 (CD86^+^) macrophages in each group. (E) Relative messenger RNA (mRNA) expression levels of proinflammatory factors (TNF-α, iNOS, IL-1β, IL-6, and Cox-2). (F) Cytotoxicity assays of PMMs@mGC hydrogels and their components after 1, 4, and 7 d of co-culture with ADSCs. Relative expression of (G) fibrocartilage-related genes (*Acan*, *Sox9*, *Col2*, *Col1*, and α-smooth muscle actin [*α-SMA*]) and (H) corresponding proteins in ADSCs treated with TGFβ3/IGF1 composite growth factors (TI induction group), with untreated cells as the control group. Data are expressed as mean ± SD, *n* = 3. Statistical significance among various groups was determined by 1-way/2-way ANOVA and a *t* test.*P < 0.05, **P < 0.01, and ****P < 0.0001; ns, no significance. DAPI, 4′,6-diamidino-2-phenylindole; Ctrl, control; Exp, experimental.

Macrophages are known to act as immune defenders by eliminating proinflammatory cytokines, thereby exerting immunoregulatory effects that support tissue regeneration [55]. We hypothesize that PMMs can similarly self-eliminate proinflammatory cytokines via electrostatic interactions and sequentially regulate fibrocartilage regeneration, functioning as macrophage-mimicking nanomaterials. As shown in Fig. 4A, BPEI-modified PMMs with an electropositive shell selectively adsorb electronegative proinflammatory factors (e.g., IL-1β, IL-6, and TNF-α) and subsequently dissociate from the mesoporous silica core, allowing adequate clearance of the bound cytokines via metabolic processes. Following this, the TGFβ3/IGF1 composite growth factors encapsulated in the core are promptly released to induce ADSC differentiation into fibrochondrocytes, triggered by the dissociation of the modified outer shell. Before investigating these biological functions, cytocompatibility was first assessed using the Cell Counting Kit-8 (CCK-8) assay, live/dead staining, and F-actin staining. As shown in Fig. 4B and Fig. S3, ADSCs encapsulated in PMMs@mGC hydrogels demonstrated high cell viability and well-defined cytoskeletal structures after 7 d of culture, indicating that the hydrogel scaffold provides a favorable environment for 3D cell culture and tissue regeneration. Additionally, the CCK-8 assay confirmed that PMMs@mGC hydrogel extracts and their components exhibited no significant cytotoxicity after 1, 4, and 7 d of co-culture with ADSCs (Fig. 4F).

Furthermore, the anti-inflammatory effects of PMMs were assessed by polarizing macrophages using lipopolysaccharide (LPS) induction, followed by co-culture with BPEI or PMM extracts. Flow cytometry results showed that both BPEI and PMMs suppressed M1 macrophage polarization compared with the LPS group, with the PMMs group exhibiting the lowest polarization rate (Fig. 4C). Quantitative analysis further confirmed that PMMs effectively inhibited the proinflammatory M1 phenotype (Fig. 4D). As previously reported, meniscal injury often triggers the release of key inflammatory cytokines, including TNF-α, IL-1β, IL-6, and COX-2 [30,56]. The cytokine adsorption and clearance capabilities of the macrophage-mimicking nanomaterials were verified by quantitative real-time polymerase chain reaction (qPCR). The LPS-induced group showed the highest messenger RNA expression levels of *TNF-α*, *INOS*, *IL-1β*, *IL-6*, and *Cox-2*, whereas both the BPEI-treated and PMM-treated groups showed significantly reduced expression levels. This reduction is likely due to efficient removal of proinflammatory cytokines (Fig. 4E). Notably, the reduction observed in qPCR was more pronounced than that seen in flow cytometry, likely due to the physical adsorption of electronegative cytokines by electropositive BPEI, rather than direct inhibition of inflammation. After confirming the cytocompatibility and cytokine clearance ability of PMMs, their fibrochondrogenic induction potential was further evaluated by qPCR and western blot. In these experiments, ADSCs treated with the TGFβ3/IGF1 composite growth factors served as the TI induction group, while untreated ADSCs served as the control. After 28 d of culture, both qPCR and western blot results showed significantly elevated expression levels of fibrocartilage-related genes and proteins (*Acan*, *Sox9*, *Col2*, *Col1*, and α-smooth muscle actin [*α-SMA*]) in the TI group compared with the control (Fig. 4G and H). These findings confirm that the combined use of TGFβ3 and IGF1 efficiently induces ADSC differentiation into fibrochondrocytes, as evidenced by the upregulation of fibrochondrogenic markers. Overall, macrophage-mimicking nanomaterials demonstrate high cytocompatibility, autonomous clearance of proinflammatory cytokines, and induction of fibrocartilage differentiation. These results support their ability to modulate the stepwise healing cascade from inflammation resolution to meniscal fibrocartilage regeneration.

### In vivo evaluation of inflammatory meniscus defect repair

**Fig. 5.**
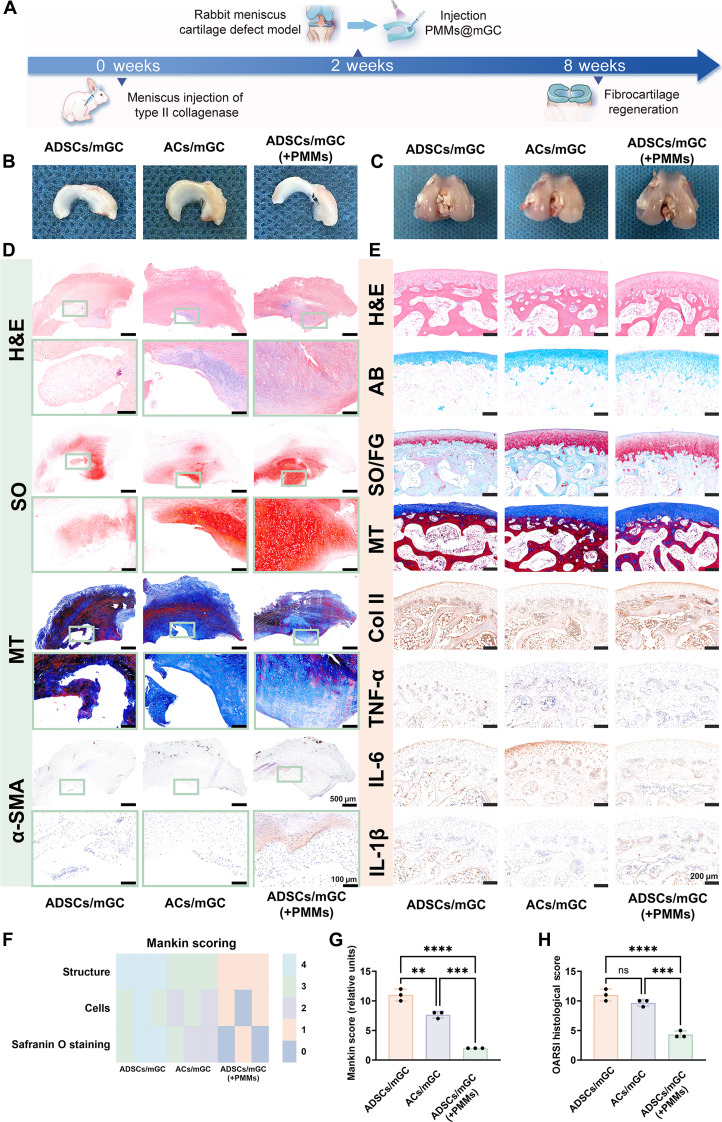
In vivo evaluation of inflammatory meniscus defect repair. (A) Schematic illustration of meniscus defect repair using ADSC-loaded PMMs@mGC hydrogels in a rabbit osteoarthritis model. Representative gross view of (B) meniscus surfaces and (C) femoral cartilage surfaces at 8 weeks postsurgery across different groups: ADSCs/mGC, ACs/mGC, and ADSCs/mGC(+PMMs). (D) Histological staining by H&E, Safranin O (SO), and MT, along with immunohistochemical staining for α-SMA in repaired meniscus regions. (E) Histological staining by H&E, Alcian Blue (AB), SO/Fast Green (SO/FG), and MT, along with immunohistochemical staining for COL II, TNF-α, IL-6, and IL-1β in repaired femoral cartilage regions. (F) Heatmap and (G) histogram of Mankin scores for each group. (H) Histogram of Osteoarthritis Research Society International (OARSI) scores for each group. Data are expressed as mean ± SD, *n* = 3. Statistical significance among groups was determined using one-way ANOVA and a *t* test. **P < 0.01, ***P < 0.001, and ****P < 0.0001; ns, no significance.

To further investigate the feasibility of repairing inflammatory meniscus defects, a rabbit OA model was first established in the knee joint cavity, following a previously reported method [57,58] involving intra-articular injection of a type II collagenase solution (1 ml of saline containing 8 mg of type II collagenase). Two weeks after inducing early inflammation in the articular cavity, a 2-mm-diameter cylindrical defect was created in the meniscus using a trephine instrument. Meniscus defects were then repaired by injecting seed-cell-loaded gel precursors, which were rapidly cross-linked under light irradiation (365-nm LED, 20 mW/cm^2^) to form hydrogel scaffolds (Fig. 5A and Fig. S4). In this study, meniscus defect repair was evaluated across 3 groups: (a) auricular-chondrocyte-loaded mGC hydrogels (ACs/mGC), (b) ADSC-loaded mGC hydrogels (ADSCs/mGC), and (c) ADSC-loaded PMMs@mGC hydrogels (ADSCs/mGC(+PMMs)). At 8 weeks postsurgery, both repaired meniscus and articular tissue samples were collected for further analysis. As shown in Fig. 5B, gross examination revealed nearly complete macroscopic healing in the ADSCs/mGC(+PMMs) group. By contrast, visible cartilage defects remained in both the ACs/mGC and ADSCs/mGC groups, likely due to the harsh inflammatory environment within the joint cavity. On the femoral cartilage surface, OA-related damage was evident in the ACs/mGC and ADSCs/mGC groups. In contrast, the ADSCs/mGC(+PMMs) group showed a near-normal appearance, attributed to PMMs’ self-clearance of proinflammatory factors (Fig. 5C).

Histological and immunohistochemical analyses were performed to assess both meniscus regeneration and joint inflammation suppression. As shown in Fig. 5D and Fig. S5, the ADSCs/mGC(+PMMs) group exhibited continuous tissue regeneration and fibrocartilage-specific matrix deposition, with positive cartilage staining in H&E and Safranin O, as well as fibrous tissue staining in MT and α-SMA. By contrast, both the ACs/mGC and ADSCs/mGC groups showed pronounced tissue deficiency, consistent with the inflammatory conditions of the OA model. Further evaluation of the articular cartilage surface, including immunohistochemical staining for TNF-α, IL-6, and IL-1β, revealed that the ADSCs/mGC(+PMMs) group had the lowest levels of proinflammatory cytokines compared with the other 2 groups (Fig. 5E). This suggests that PMMs effectively cleared proinflammatory factors in vivo. Notably, this suppression of inflammation also supported cartilage repair, as evidenced by positive staining for cartilage-specific matrix markers, including Alcian Blue (AB), SO, and COL II. Figure 5F and G show the Mankin scores and corresponding histograms for each group. Statistical analysis demonstrated that the ADSCs/mGC group had the highest scores, indicating severe cartilage damage, while the ADSCs/mGC(+PMMs) group had the lowest scores, reflecting strong protective effects. Similarly, Osteoarthritis Research Society International (OARSI) scores confirmed the superior repair efficacy of the ADSCs/mGC(+PMMs) group (Fig. 5H). Therefore, PMMs@mGC hydrogels effectively eliminated proinflammatory cytokines and protected articular cartilage from inflammatory injury in vivo. More importantly, these findings illustrate the success of a cascade regulation strategy that transitions from inflammation elimination to meniscal fibrocartilage regeneration.

### In vivo evaluation of inflammatory total meniscus replacement

**Fig. 6.**
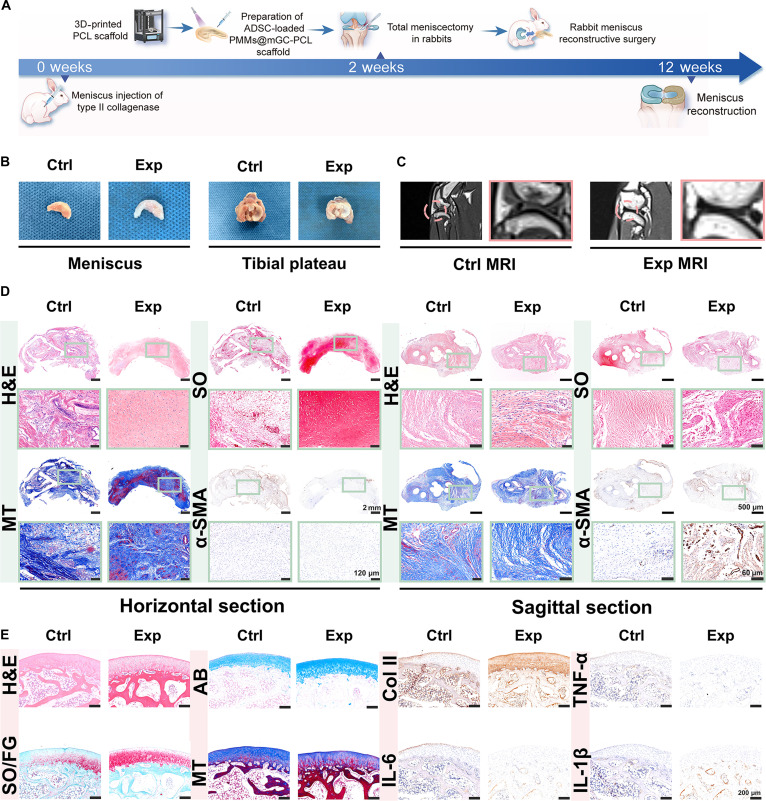
In vivo evaluation of inflammatory total meniscus replacement. (A) Schematic illustration of total meniscus replacement using ADSC-loaded PMMs@mGC-PCL scaffolds in a rabbit osteoarthritis model. Representative (B) gross view and (C) magnetic resonance imaging of meniscus and femoral cartilage surfaces at 12 weeks postsurgery in each group. ADSC-loaded PMMs@mGC-PCL scaffolds serve as the Exp group; PCL-only scaffolds serve as the Ctrl group. (D) Histological staining by H&E, SO, and MT, along with immunohistochemical staining for α-SMA in horizontal and sagittal sections for each group. (E) Histological staining by H&E, AB, SO/FG, and MT, along with immunohistochemical staining for COL II, TNF-α, IL-6, and IL-1β.

Beyond small-sized meniscus defect repair, larger injuries typically require total meniscus replacement. To investigate the feasibility of inflammatory total meniscus replacement, a rabbit OA model was established as before, using intra-articular injection of type II collagenase solution (1 ml of saline with 8 mg of type II collagenase). To enable total meniscus replacement, we adopted a “steel–concrete” tissue-engineering strategy by combining 3D-printed crescent-shaped PCL frameworks as the “steel” mechanical support with ADSC-loaded mGC(+PMMs) hydrogels as the “concrete” regenerative component (Fig. 6A and Fig. S5). Two weeks after OA induction, the native meniscus was surgically removed and replaced with a crescent-shaped ADSC-loaded PMMs@mGC-PCL scaffold in the experimental (Exp) groups. In contrast, the control (Ctrl) groups received PCL scaffolds alone. At 12 and 16 weeks postsurgery, macroscopic observation revealed that the Exp groups closely resembled the native meniscus in both crescent shape and fibrocartilage-like texture (Fig. 6B and Fig. S6). By contrast, the Ctrl groups showed a softer, fibrous-like tissue with residual PCL material. Magnetic resonance imaging confirmed that the Exp groups maintained both crescent morphology and signal intensity comparable to that of the native meniscus, whereas the Ctrl groups lacked clear evidence of meniscus regeneration (Fig. 6C).

Histological and immunohistochemical analyses were conducted to evaluate meniscal tissue reconstruction in horizontal and sagittal planes. The staining panel comprised H&E, AB, SO with Fast Green, and MT. As shown in Fig. 6D and Fig. S7, the Exp groups exhibited fibrocartilage-like features, as evidenced by positive GAG and collagen fiber staining. In contrast, the Ctrl groups displayed disorganized fibrous tissue and lacked cartilage-related architecture. Notably, sagittal sections of the regenerated meniscus in the Exp groups revealed heterogeneous tissue structure and integration, whereas the Ctrl groups exhibited larger voids in the central regions. These results suggest that the ADSC-loaded PMMs@mGC-PCL scaffold effectively restored the morphology and function of the native meniscus. In addition to meniscal reconstruction, articular cartilage conditions and inflammatory cytokine levels were evaluated. As shown in Fig. 6E, histological and immunohistochemical analyses indicate that the PMMs@mGC-PCL scaffold preserves articular cartilage surface integrity, with a more pronounced effect in the lateral compartment. In contrast, the PCL scaffold alone is associated with greater cartilage wear, likely attributable to friction from the rigid scaffold. Furthermore, the expression levels of TNF-α, IL-6, and IL-1β were lower in the PMMs@mGC-PCL group than in the PCL-alone group, reflecting PMMs’ adequate cytokine clearance in the OA model. Mankin scoring heatmaps and histograms, along with OARSI scoring results, confirmed the optimal regenerative outcome in the Exp groups (Fig. S8). Overall, the ADSC-loaded PMMs@mGC-PCL scaffolds successfully reconstructed the meniscus, restored functional integrity, and provided significant protection to the articular cartilage by effectively regulating inflammation. These results underscore the strong therapeutic potential of our “steel–concrete” tissue-engineering strategy for total meniscus reconstruction and joint preservation.

## Conclusion

In summary, this study presents a macrophage-mimicking nanomaterial capable of cascade regulation for inflammatory meniscus therapy, facilitating both the clearance of proinflammatory cytokines and fibrocartilage regeneration. The carefully designed PMMs effectively eliminate proinflammatory factors via physical adsorption and enable the timely release of TGFβ3 and IGF1, thereby promoting ADSC differentiation into fibrochondrocytes within the inflammatory environment. For therapeutic application, ADSC-loaded PMMs@mGC hydrogels demonstrated satisfactory outcomes in meniscus defect repair and fibrocartilage regeneration. Moreover, total meniscus replacement was successfully achieved by combining a crescent-shaped PCL framework with a “steel–concrete” tissue-engineering strategy. Naturally, several issues remain to be addressed before clinical translation, including the evaluation of nanomaterial biosafety, validation in large animal models, and the development of standardized production protocols. Overall, this innovative macrophage-mimicking material holds strong clinical potential for both meniscal injury repair and total meniscus replacement in the context of OA.

## Materials and Methods

### Materials and animals

Unless otherwise stated, all chemicals used in this study were of reagent grade. Dulbecco’s modified Eagle medium (DMEM) and fetal bovine serum were purchased from Gibco (USA). All other chemicals were obtained from Sigma-Aldrich (USA). Deionized water was used in all experiments. New Zealand White rabbits were procured from Shanghai Jiao Tong University Nongsheng Experimental Field Co., Ltd. (Shanghai, China).

### Fabrication of MSN_TI_-BPEI

#### Synthesis of MSNs

MSNs were synthesized according to previously reported protocols, as described as follows: First, 7.85 ml of ammonium hydroxide and 50 ml of deionized water were added to 357 ml of anhydrous ethanol with constant stirring. After 10 min, 10 ml of tetraethyl orthosilicate (TEOS) was slowly added dropwise. The mixture was then stirred for an additional hour. Simultaneously, in a separate container, 40 g of cetyltrimethylammonium chloride (CTAC) and 4 ml of triethanolamine (TEA) were dissolved in 400 ml of deionized water. The solution was stirred at room temperature for 1.5 h. The TEOS-containing solution was centrifuged at 12,000 rpm and 4 °C for 20 min. The collected precipitate was redispersed in anhydrous ethanol and centrifuged 3 additional times using the same washing protocol. After washing, the precipitate was slowly added to the CTAC–TEA solution and stirred for 1.5 h. The resulting mixture was transferred to a water bath preheated to 80 °C. Then, 3 ml of TEOS was slowly added dropwise, and the reaction was allowed to proceed for an additional hour. The mixture was cooled to room temperature and then reheated to 50 °C. At this stage, 12.72 g of sodium carbonate (Na_2_CO_3_) was added, and the mixture was stirred for 30 min. After the reaction, the mixture was centrifuged at 12,000 rpm and 4 °C for 20 min. The precipitate was washed 3 times with a sodium chloride–methanol solution. Finally, the purified product was freeze-dried and stored at 4 °C.

#### Functionalization of MSNs

The freeze-dried nanoparticles were weighed and then dispersed in deionized water. For each 1 g of nanoparticles, 2 ml of 3-mercaptopropyltrimethoxysilane was added. The suspension was stirred overnight at room temperature. After reaction completion, the mixture was centrifuged at 12,000 rpm and 4 °C for 20 min. The resulting product was then freeze-dried. The freeze-dried nanoparticles were redispersed in deionized water. For each 100 mg of nanoparticles, 10 μg of TGFβ3 and 10 μg of IGF1 were added. The suspension was sonicated in an ultrasonic bath placed in an ice bath for 30 min and then stirred overnight at room temperature. After stirring, 40 mg of BSA was added, and the suspension was stirred for a further 8 h. The mixture was centrifuged at 12,000 rpm and 4 °C for 20 min. The precipitate was collected and redispersed in deionized water. For each 100 mg of nanoparticles, 10 ml of BPEI was added. The suspension was then stirred overnight at room temperature. Finally, the product was centrifuged at 12,000 rpm and 4 °C for 20 min. The resulting precipitate was collected and freeze-dried. The final product, referred to as MSN_TI_-BPEI nanoparticles, was thus obtained.

### Characterization of MSN_TI_-BPEI

To systematically analyze the morphology, particle size, elemental composition, surface properties, and sustained-release performance of MSN_TI_-SH, MSN_TI_-BSA, and MSN_TI_-BPEI nanoparticles, the following methodologies were employed: (a) TEM and atomic force microscopy were used to observe the morphology, particle size distribution, and overall thickness of the nanoparticles. In addition, energy-dispersive x-ray spectroscopy was used to examine the elemental composition and relative elemental percentages for each nanoparticle type. (b) The zeta potential of the nanoparticles was measured using a zeta potential analyzer to evaluate their surface charge characteristics and stability. (c) Solid-state ^1^H nuclear magnetic resonance spectroscopy and Fourier transform infrared spectroscopy were employed to assess the composition, structural features, and physicochemical properties. (d) X-ray photoelectron spectroscopy was used to determine the elemental composition of the nanoparticle surfaces, providing further insight into the binding energies and chemical shifts of the constituent elements. (e) The sustained-release characteristics of TGFβ3 and IGF1 encapsulated within MSN_TI_-BPEI nanoparticles were evaluated using an enzyme-linked immunosorbent assay kit. The nanoparticles were incubated in phosphate-buffered saline (PBS) containing 0.5% dithiothreitol (Yuntian Biotechnology Co., Ltd.) at specific time points (1, 3, 4, 7, 14, 21, and 28 d). At each time point, samples were collected and analyzed in triplicate. Standard curves were generated based on the optical density values of calibration samples, and the release profiles of the growth factors were plotted over time.

### Preparation and characterization of ACMMA

In this study, fresh porcine meniscus tissue was commercially obtained and subjected to decellularization to prepare a methacrylic anhydride-modified acellular meniscus matrix (ACMMA). The detailed procedure is described as follows: Fresh porcine menisci were frozen at −80 °C and then thawed to room temperature. This freeze–thaw cycle was repeated 3 times. The thawed meniscus tissue was cut into uniform fragments approximately 1 mm × 1 mm. The fragments were immersed in deionized water and 0.1 mol/l Tris–HCl buffer (pH 7.5). The mixture was stirred at room temperature for 20 h using a magnetic stirrer.

After incubation, the mixture was centrifuged at 3,000 rpm for 10 min at 4 °C to remove the supernatant. Next, 1% (v/v) Triton X-100 in PBS was added, and the mixture was stirred at room temperature for 24 h. After stirring, the mixture was centrifuged at 3,000 rpm for 10 min at 4 °C, and the supernatant was discarded. The fragments were washed 3 times with PBS. Subsequently, 157.6 g of Tris–HCl, 16.7 mg of DNase, and 7.15 mg of RNase were sequentially dissolved in 500 ml of deionized water. This enzyme solution was mixed with the meniscus fragments and stirred at room temperature for 4 h. The mixture was then centrifuged at 3,000 rpm for 10 min at 4 °C, and the supernatant was discarded. The fragments were washed 6 times with PBS. After washing, the tissues were freeze-dried using a lyophilizer to obtain a dry acellular meniscus matrix (ACM). The freeze-dried ACM was ground into fine powder using a ball mill and then sieved through a 300-mesh screen to achieve a uniform particle size. The sieved ACM powder was weighed and digested in pepsin solution (2 mg of pepsin per 10 mg of ACM) with magnetic stirring for 24 h. After digestion, the samples were freeze-dried to obtain water-soluble ACM powder. To prepare ACMMA, 100 mg of water-soluble ACM powder was dissolved in deionized water and cooled in an ice bath. While stirring, 0.1 ml of methacrylic anhydride was slowly added dropwise.

The reaction was allowed to proceed. The pH was adjusted to 8 to 9 using 5 M NaOH, and stirring was continued overnight to ensure complete grafting of methacrylic anhydride. After the reaction, the samples were freeze-dried. The resulting ACMMA was sealed and stored at −20 °C. To evaluate the decellularization efficiency and modification status of ACMMA, histological analyses were performed on both unmodified and modified samples. Additionally, GAG content, collagen content, and residual dsDNA levels were determined. These parameters were used to evaluate the effectiveness of the decellularization process, the retention of essential matrix components, and the removal of nucleic acids.

### Preparation of PMMs@mGC hydrogels

The GelMA hydrogel was prepared following previously reported methods. Briefly, GelMA (8% w/v) was mixed with 0.25% (w/v) lithium phenyl-2,4,6-trimethylbenzoylphosphinate photoinitiator solution, which had been pre-dissolved in deionized water. The mixture was stirred at room temperature until fully dissolved to form the GelMA hydrogel. The hydrogel was then stored at −20 °C until further use.

To fabricate the mGC hydrogel, the GelMA hydrogel (8% w/v) was homogeneously mixed with 1% (w/v) methacrylated decellularized meniscus extracellular matrix (ACMMA). The PMMs@mGC hydrogel was prepared by adding 1% (w/v) MSN_TI_-BPEI to the mGC hydrogel (containing 8% w/v GelMA and 1% w/v ACMMA), followed by thorough mixing. The resulting PMMs@mGC hydrogel was used for subsequent experimental analyses.

### Characterization of PMMs@mGC

The hydrogels were categorized into 2 groups: the ACMMA group (mGC) and the nanoparticle group (PMMs@mGC). The following analyses were performed: the physical states of the hydrogels were observed before and after photo-cross-linking. After freeze-drying, microstructural differences between the 2 groups were examined by scanning electron microscopy. The mechanical properties of the photo-cross-linked hydrogels were assessed by recording force–displacement curves. Storage modulus values were presented as bar graphs. Changes in storage modulus (*G*′) and loss modulus (*G*″) before and after photo-cross-linking were measured using a rheometer. The water contact angle of the photo-cross-linked hydrogels was calculated to assess surface wettability. Swelling and degradation behaviors were evaluated using a gravimetric method, as previously described in the literature. The initial wet weight (*W*_0_) and the wet weight at various time points (*W*_s_) were recorded. The swelling ratio was calculated as (*W*_s_/*W*_0_) × 100%. For the degradation test, the initial wet weight (*W*_d_) and the remaining wet weight after incubation in 1% (w/v) collagenase solution (*W*_t_) were recorded. The degradation rate was calculated as (*W*_t_/*W*_d_) × 100%.

### Cell culture

In this study, subcutaneous inguinal adipose tissue and auricular cartilage from New Zealand White rabbits were used as sources of cells. Fresh adipose tissue was finely minced and digested in 0.15% type II collagenase at 37 °C for 1 h to isolate ADSCs. The isolated cells were cultured and expanded in low-glucose DMEM supplemented with 5% fetal bovine serum and 1% penicillin–streptomycin at 37 °C in a humidified atmosphere containing 5% CO_2_. Auricular cartilage was carefully dissected to remove the perichondrium, finely minced, and digested in 0.15% type II collagenase at 37 °C for 24 h to isolate chondrocytes.

### Cytotoxicity assessment

Cells were seeded at a density of 1,000 cells per well into a 24-well culture plate. The hydrogels were divided into 3 groups, control, mGC, and PMMs@mGC, and each group was co-cultured with ADSCs. Five replicate wells were prepared for each group. At each time point (days 1, 4, and 7), 30 μl of CCK-8 reagent was added to each well. The plates were incubated in the dark for 2 h. Absorbance was then measured at 450 nm using a microplate reader. For hydrogel preparation, passage 2 ADSCs were mixed with either mGC or PMMs@mGC hydrogels at a 1:9 (v/v) ratio. The mixtures were placed into cylindrical molds (9 mm in diameter, 2.5 mm in height) and cross-linked under a curing lamp (365-nm LED, 20 mW/cm^2^) for 30 s.

### Anti-inflammatory assay

RAW264.7 cells (2 × 10^5^ per well) were seeded into a 6-well culture plate. The cells were stimulated with 1 μg/ml LPS for 12 h. Subsequently, they were incubated with BPEI and PMMs@mGC for an additional 24 h. After incubation, the cells were collected and stained with CD86 and CD68 antibodies. Flow cytometry was performed to analyze the expression of CD86 and CD68. Cells were collected by repeating the above procedures. Total RNA was extracted using a commercial kit and then reverse-transcribed. Gene expression was quantified using SYBR Green-based qPCR. The expression levels of inflammation-related genes (*TNF-α*, *iNOS*, *IL-1β*, *IL-6*, and *Cox-2*) were calculated using the 2^−ΔΔCT^ method.

### In vitro chondrogenic induction of ADSCs

ADSCs were seeded at a density of 3 × 10^5^ cells per well and maintained in a standard cell culture incubator. After 3 d, the medium was replaced with chondrogenic induction medium supplemented with 10 ng/ml TGFβ3 and 10 ng/ml IGF1. The induction medium was refreshed every 3 d for a total duration of 28 d. On day 28, cells were harvested. The expression levels of chondrocyte-related genes (*Acan*, *Col1*, *Col2*, *Sox9*, and *α-SMA*) in both noninduced ADSCs and chondrogenically induced ADSCs were evaluated by qPCR and western blot analysis.

### Repair of the rabbit inflammatory meniscus defect model

In this experiment, healthy 12-week-old New Zealand White rabbits were used. An early meniscus inflammation model was established by injecting 0.5 ml of type II collagenase solution (4 mg/ml in PBS) into the right knee joint cavity, followed by a 2-week incubation period. The animals were randomly divided into 3 groups: ADSCs/mGC, ACs/mGC, and ADSCs/mGC(+PMMs). After 2 weeks, cell-loaded hydrogel scaffolds were prepared. The animals then underwent meniscus defect modeling followed by in situ hydrogel formation. Tissue samples were collected at 4 and 8 weeks postsurgery for further analysis.

### Rabbit inflammatory total meniscectomy and replacement model

An early meniscus inflammation model was established according to previously published protocols. The normal rabbit meniscus was reconstructed and modeled in 3 dimensions. A porous PCL scaffold was fabricated using 3D printing. The scaffold was then coated with PMMs@mGC hydrogels loaded with ADSCs and subsequently photo-cross-linked. The experimental animals were randomly assigned to 2 groups: control (Ctrl) and experimental (Exp). After total meniscectomy, each group underwent total meniscal replacement using either the PCL scaffold or the PMMs@mGC hydrogel scaffold loaded with ADSCs. Tissue samples were collected at 6 and 12 weeks postsurgery for further analysis.

### Histological and immunohistochemical analysis

Meniscus tissue was fixed in 4% paraformaldehyde for 3 d, followed by paraffin embedding. Joint tissue was fixed in 4% paraformaldehyde for 3 d, decalcified in a decalcifying solution for 2 weeks, and then embedded in paraffin. After sectioning, tissue samples were stained with H&E, SO, MT, and α-SMA to evaluate meniscus regeneration. The articular cartilage surface was stained with H&E, SO, AB, and MT to assess cartilage surface damage. Additionally, immunohistochemical staining for COL I, COL II, IL-1, and IL-6 was performed on the articular cartilage surface to further evaluate tissue damage and inflammatory responses. Finally, the Mankin and OARSI histological scoring systems were used for comprehensive assessment.

### Statistical analysis

Unless otherwise specified, all experiments were conducted in triplicate. Statistical analyses and data visualization were performed using the GraphPad Prism software (version 10.0). Data are expressed as mean ± standard deviation. Statistical significance was set as follows: *P < 0.05; **P < 0.01; ***P < 0.005; ****P < 0.001; and ns, no significance.

## Ethical Approval

All animal procedures were approved by the Animal Care and Laboratory Committee of Shanghai Jiao Tong University School of Medicine (approval number: SH9H-2021-A655-SB).

## Data Availability

The authors declare that all data supporting the results in this study are available within the paper and its Supplementary Materials or from the corresponding authors upon reasonable request.

## References

[1] Ding G, He Y, Shi Y, Maimaitimin M, Zhang X, Huang H, Huang W, Yu R, Wang J. Sustained-drug-release, strong, and anti-swelling water-lipid biphasic hydrogels prepared via digital light processing 3D printing for protection against osteoarthritis: Demonstration in a porcine model. Adv Healthc Mater. 2023;12(18):2203236. 10.1002/adhm.202203236 36943891

[2] Guo W, Chen M, Wang Z, Tian Y, Zheng J, Gao S, Li Y, Zheng Y, Li X, Huang J, et al. 3D-printed cell-free PCL–MECM scaffold with biomimetic micro-structure and micro-environment to enhance in situ meniscus regeneration. Bioact Mater. 2021;6(10):3620–3633. 33869902 10.1016/j.bioactmat.2021.02.019 PMC8039774

[3] Pan X, Li R, Li W, Sun W, Yan Y, Xiang X, Fang J, Liao Y, Xie C, Wang X, et al. Silk fibroin hydrogel adhesive enables sealed-tight reconstruction of meniscus tears. Nat Commun. 2024;15(1):2651. 38531881 10.1038/s41467-024-47029-6 PMC10966011

[4] Kwon H, Brown WE, Lee CA, Wang D, Paschos N, Hu JC, Athanasiou KA. Surgical and tissue engineering strategies for articular cartilage and meniscus repair. Nat Rev Rheumatol. 2019;15(9):550–570. 31296933 10.1038/s41584-019-0255-1 PMC7192556

[5] Xia B, Kim DH, Bansal S, Bae Y, Mauck RL, Heo SJ. Development of a decellularized meniscus matrix-based nanofibrous scaffold for meniscus tissue engineering. Acta Biomater. 2021;128:175–185. 33823327 10.1016/j.actbio.2021.03.074 PMC8474106

[6] Wang W, Chu Y, Lu Y, Xu J, Zhao W, Liang Z, Guo X, Xi L, Han T, Shen Y, et al. Skatole alleviates osteoarthritis by reprogramming macrophage polarization and protecting chondrocytes. Research. 2025;8:0604. 39902346 10.34133/research.0604 PMC11788598

[7] Du M, Liu K, Lai H, Qian J, Ai L, Zhang J, Yin J, Jiang D. Functional meniscus reconstruction with biological and biomechanical heterogeneities through topological self-induction of stem cells. Bioact Mater. 2024;36:358–375. 38496031 10.1016/j.bioactmat.2024.03.005 PMC10944202

[8] Shao C, Liu Y, Chi J, Ye F, Zhao Y. Hierarchically inverse opal porous scaffolds from droplet microfluidics for biomimetic 3D cell co-culture. Engineering. 2021;7(12):1778–1785.

[9] Jia L, Hua Y, Zeng J, Liu W, Wang D, Zhou G, Liu X, Jiang H. Bioprinting and regeneration of auricular cartilage using a bioactive bioink based on microporous photocrosslinkable acellular cartilage matrix. Bioact Mater. 2022;16:66–81. 35386331 10.1016/j.bioactmat.2022.02.032 PMC8958552

[10] Bai B, Liu Y, Huang J, Wang S, Chen H, Huo Y, Zhou H, Liu Y, Feng S, Zhou G, et al. Tolerant and rapid endochondral bone regeneration using framework-enhanced 3D biomineralized matrix hydrogels. Adv Sci. 2024;11(9):2305580. 10.1002/advs.202305580 PMC10916654 38127989

[11] Sun Y, Huo Y, Ran X, Chen H, Pan Q, Chen Y, Zhang Y, Ren W, Wang X, Zhou G, et al. Instant trachea reconstruction using 3D-bioprinted C-shape biomimetic trachea based on tissue-specific matrix hydrogels. Bioact Mater. 2023;32:52–65. 37818289 10.1016/j.bioactmat.2023.09.011 PMC10562117

[12] Chang Z, Ran X, Chu Y, Li B, Fan Z, Li G, Li D, Ren W, Hua Y, Zhou G. Dynamic-covalent hybrid hydrogels with cartilaginous immune microenvironment temporally regulating meniscus regeneration. Bioact Mater. 2025;50:14–29. 40242503 10.1016/j.bioactmat.2025.03.026 PMC11998115

[13] Shi T, Zhao J, Long K, Gao M, Chen F, Chen X, Zhang Y, Huang B, Shao D, Yang C, et al. Cationic mesoporous silica nanoparticles alleviate osteoarthritis by targeting multiple inflammatory mediators. Biomaterials. 2023;303: Article 122366. 37948854 10.1016/j.biomaterials.2023.122366

[14] Ghouri A, Muzumdar S, Barr AJ, Robinson E, Murdoch C, Kingsbury SR, Conaghan PG. The relationship between meniscal pathologies, cartilage loss, joint replacement and pain in knee osteoarthritis: A systematic review. Osteoarthr Cartil. 2022;30(10):1287–1327. 10.1016/j.joca.2022.08.002 35963512

[15] Liu L, Xian Y, Wang W, Huang L, Fan J, Ma W, Li Y, Liu H, Yu JK, Wu D. Meniscus-inspired self-lubricating and friction-responsive hydrogels for protecting articular cartilage and improving exercise. ACS Nano. 2023;17(23):24308–24319. 37975685 10.1021/acsnano.3c10139

[16] Moradi L, Vasei M, Dehghan MM, Majidi M, Mohajeri SF, Bonakdar S. Regeneration of meniscus tissue using adipose mesenchymal stem cells-chondrocytes co-culture on a hybrid scaffold: In vivo study. Biomaterials. 2017;126:18–30. 28242519 10.1016/j.biomaterials.2017.02.022

[17] Yuan Z, Liu S, Hao C, Guo W, Gao S, Wang M, Chen M, Sun Z, Xu Y, Wang Y, et al. AMECM/DCB scaffold prompts successful total meniscus reconstruction in a rabbit total meniscectomy model. Biomaterials. 2016;111:13–26. 27718449 10.1016/j.biomaterials.2016.09.017

[18] Wang Y, Ding H, Bai R, Li Q, Ren B, Lin P, Li C, Chen M, Xu X. Exosomes from adipose-derived stem cells accelerate wound healing by increasing the release of IL-33 from macrophages. Stem Cell Res Ther. 2025;16(1):80. 39984984 10.1186/s13287-025-04203-x PMC11846291

[19] Vélez-Pinto JF, Garcia-Arranz M, García-Bernal D, García Gómez-Heras S, Villarejo-Campos P, García-Hernández AM, Vega-Clemente L, Jiménez-Galanes S, Guadalajara H, Moraleda JM, et al. Therapeutic effect of adipose-derived mesenchymal stem cells in a porcine model of abdominal sepsis. Stem Cell Res Ther. 2023;14(1):365. 38087374 10.1186/s13287-023-03588-x PMC10717819

[20] Zhang Y, Wang S, Yang Y, Zhao S, You J, Wang J, Cai J, Wang H, Wang J, Zhang W, et al. Scarless wound healing programmed by core-shell microneedles. Nat Commun. 2023;14(1):3431. 37301874 10.1038/s41467-023-39129-6 PMC10257705

[21] Su W, Yu S, Yin Y, Li B, Xue J, Wang J, Gu Y, Zhang H, Lyu Z, Mu Y, et al. Diabetic microenvironment preconditioning of adipose tissue-derived mesenchymal stem cells enhances their anti-diabetic, anti-long-term complications, and anti-inflammatory effects in type 2 diabetic rats. Stem Cell Res Ther. 2022;13(1):422. 35986406 10.1186/s13287-022-03114-5 PMC9389728

[22] Ouyang L, Qiu D, Fu X, Wu A, Yang P, Yang Z, Wang Q, Yan L, Xiao R. Overexpressing HPGDS in adipose-derived mesenchymal stem cells reduces inflammatory state and improves wound healing in type 2 diabetic mice. Stem Cell Res Ther. 2022;13(1):395. 35922870 10.1186/s13287-022-03082-w PMC9351105

[23] Zhao B, Han J, Hu D. Letter to the editor regarding microneedle-mediated biomimetic cyclodextrin metal organic frameworks for active targeting and treatment of hypertrophic scars. ACS Nano. 2022;16(6):8507–8508. 10.1021/acsnano.1c07829 34792332

[24] Wu T, Hou X, Li J, Ruan H, Pei L, Guo T, Wang Z, Ci T, Ruan S, He Y, et al. Microneedle-mediated biomimetic cyclodextrin metal organic frameworks for active targeting and treatment of hypertrophic scars. ACS Nano. 2021;15(12):20087–20104. 34792332 10.1021/acsnano.1c07829

[25] Correa S, Grosskopf AK, Lopez Hernandez H, Chan D, Yu AC, Stapleton LM, Appel EA. Translational applications of hydrogels. Chem Rev. 2021;121(18):11385–11457. 33938724 10.1021/acs.chemrev.0c01177 PMC8461619

[26] Tian M, Ticer T, Wang Q, Walker S, Pham A, Suh A, Busatto S, Davidovich I, Al-Kharboosh R, Lewis-Tuffin L, et al. Adipose-derived biogenic nanoparticles for suppression of inflammation. Small. 2020;16(10):1904064. 10.1002/smll.201904064 32067382

[27] Mao X, Cheng R, Zhang H, Bae J, Cheng L, Zhang L, Deng L, Cui W, Zhang Y, Santos HA, et al. Self-healing and injectable hydrogel for matching skin flap regeneration. Adv Sci. 2019;6(3):1801555. 10.1002/advs.201801555 PMC6364594 30775235

[28] Liu X, Guo Z, Wang J, Shen W, Jia Z, Jia S, Li L, Wang J, Wang L, Li J, et al. Thiolation-based protein–protein hydrogels for improved wound healing. Adv Healthc Mater. 2024;13(14):2303824. 10.1002/adhm.202303824 38303578

[29] Eweje F, Walsh ML, Ahmad K, Ibrahim V, Alrefai A, Chen J, Chaikof EL. Protein-based nanoparticles for therapeutic nucleic acid delivery. Biomaterials. 2024;305: Article 122464. 38181574 10.1016/j.biomaterials.2023.122464 PMC10872380

[30] Zhong G, Yao J, Huang X, Luo Y, Wang M, Han J, Chen F, Yu Y. Injectable ECM hydrogel for delivery of BMSCs enabled full-thickness meniscus repair in an orthotopic rat model. Bioact Mater. 2020;5(4):871–879. 32637750 10.1016/j.bioactmat.2020.06.008 PMC7332471

[31] Pang Q, Chen Z, Li X, Zhan J, Huang W, Lei Y, Bao W. Cytokine-activated mesenchymal-stem-cell-derived extracellular matrix facilitates cartilage repair by enhancing chondrocyte homeostasis and chondrogenesis of recruited stem cells. Research. 2025;8:0700. 41049612 10.34133/research.0700 PMC12494090

[32] Li J, Fan J, Gao Y, Huang S, Huang D, Li J, Wang X, Santos HA, Shen P, Xia B. Porous silicon nanocarriers boost the immunomodulation of mitochondria-targeted bovine serum albumins on macrophage polarization. ACS Nano. 2023;17(2):1036–1053. 36598186 10.1021/acsnano.2c07439 PMC9878978

[33] Visalakshan RM, Bright R, Burzava AL, Barker AJ, Simon J, Ninan N, Palms D, Wood J, Martínez-Negro M, Morsbach S, et al. Antibacterial nanostructured surfaces modulate protein adsorption, inflammatory responses, and fibrous capsule formation. ACS Appl Mater Interfaces. 2023;15(1):220–235. 36416784 10.1021/acsami.2c13415

[34] Yan W, Cheng J, Wu H, Gao Z, Li Z, Cao C, Meng Q, Wu Y, Ren S, Zhao F, et al. Vascular smooth muscle cells transdifferentiate into chondrocyte-like cells and facilitate meniscal fibrocartilage regeneration. Research. 2024;7:0555. 39717465 10.34133/research.0555 PMC11665451

[35] Shen J, Chen A, Cai Z, Chen Z, Cao R, Liu Z, Li Y, Hao J. Exhausted local lactate accumulation via injectable nanozyme-functionalized hydrogel microsphere for inflammation relief and tissue regeneration. Bioact Mater. 2022;12:153–168. 35310385 10.1016/j.bioactmat.2021.10.013 PMC8897073

[36] Lee J, Lee S, Huh SJ, Kang BJ, Shin H. Directed regeneration of osteochondral tissue by hierarchical assembly of spatially organized composite spheroids. Adv Sci. 2022;9(3):2103525. 10.1002/advs.202103525 PMC8787388 34806336

[37] Cheng L, Cai Z, Ye T, Yu X, Chen Z, Yan Y, Qi J, Wang L, Liu Z, Cui W, et al. Injectable polypeptide-protein hydrogels for promoting infected wound healing. Adv Funct Mater. 2020;30(25):2001196.

[38] Dawulieti J, Sun M, Zhao Y, Shao D, Yan H, Lao YH, Hu H, Cui L, Lv X, Liu F, et al. Treatment of severe sepsis with nanoparticulate cell-free DNA scavengers. Sci Adv. 2020;6(22): Article eaay7148. 32523983 10.1126/sciadv.aay7148 PMC7259927

[39] Gu P, Wusiman A, Wang S, Zhang Y, Liu Z, Hu Y, Liu J, Wang D. Polyethylenimine-coated PLGA nanoparticles-encapsulated Angelica sinensis polysaccharide as an adjuvant to enhance immune responses. Carbohydr Polym. 2019;223: Article 115128. 31427012 10.1016/j.carbpol.2019.115128

[40] Zhang X, Yu Y, Shen J, Qi W, Wang H. Fabrication of polyethyleneimine-functionalized reduced graphene oxide-hemin-bovine serum albumin (PEI-rGO-hemin-BSA) nanocomposites as peroxidase mimetics for the detection of multiple metabolites. Anal Chim Acta. 2019;1070:80–87. 31103170 10.1016/j.aca.2019.04.028

[41] Liu Z, Chen X, Zhang Z, Zhang X, Saunders L, Zhou Y, Ma PX. Nanofibrous spongy microspheres to distinctly release miRNA and growth factors to enrich regulatory T cells and rescue periodontal bone loss. ACS Nano. 2018;12(10):9785–9799. 30141906 10.1021/acsnano.7b08976 PMC6205210

[42] Tang F, Li L, Chen D. Mesoporous silica nanoparticles: Synthesis, biocompatibility and drug delivery. Adv Mater. 2012;24(12):1504–1534. 22378538 10.1002/adma.201104763

[43] Li H, Zhao T, Yuan Z, Gao T, Yang Y, Li R, Tian Q, Tang P, Guo Q, Zhang L. Cartilage lacuna-biomimetic hydrogel microspheres endowed with integrated biological signal boost endogenous articular cartilage regeneration. Bioact Mater. 2024;41:61–82. 39104774 10.1016/j.bioactmat.2024.06.037 PMC11299526

[44] Zhao D, Li Y, Xiang L, Saiding Q, Lin Z, Cai Z, Wang J, Cui W. Cell shock absorption via stress relaxation hydrogel microspheres for alleviating endoplasmic reticulum stress in chondrocytes. Research. 2025;8:0777. 40678150 10.34133/research.0777 PMC12267986

[45] Xiong W, Han Z, Ding S, Wang H, Du Y, Cui W, Zhang M. In situ remodeling of efferocytosis via lesion-localized microspheres to reverse cartilage senescence. Adv Sci. 2024;11(19):2400345. 10.1002/advs.202400345 PMC11109622 38477444

[46] Dou H, Wang S, Hu J, Song J, Zhang C, Wang J, Xiao J. Osteoarthritis models: From animals to tissue engineering. J Tissue Eng. 2023;14:20417314231172584. 37223125 10.1177/20417314231172584 PMC10201005

[47] Li Z, Wu N, Cheng J, Sun M, Yang P, Zhao F, Zhang J, Duan X, Fu X, Zhang J, et al. Biomechanically, structurally and functionally meticulously tailored polycaprolactone/silk fibroin scaffold for meniscus regeneration. Theranostics. 2020;10(11):5090. 32308770 10.7150/thno.44270 PMC7163455

[48] Guo S, He C. Bioprinted scaffold remodels the neuromodulatory microenvironment for enhancing bone regeneration. Adv Funct Mater. 2023;33(40):2304172.

[49] Fang A, Wang Y, Guan N, Zuo Y, Lin L, Guo B, Mo A, Wu Y, Lin X, Cai W, et al. Porous microneedle patch with sustained delivery of extracellular vesicles mitigates severe spinal cord injury. Nat Commun. 2023;14(1):4011. 37419902 10.1038/s41467-023-39745-2 PMC10328956

[50] Pardo A, Gomez-Florit M, Barbosa S, Taboada P, Domingues RM, Gomes ME. Magnetic nanocomposite hydrogels for tissue engineering: Design concepts and remote actuation strategies to control cell fate. ACS Nano. 2021;15(1):175–209. 33406360 10.1021/acsnano.0c08253

[51] Zhu H, Yang H, Ma Y, Lu TJ, Xu F, Genin GM, Lin M. Spatiotemporally controlled photoresponsive hydrogels: Design and predictive modeling from processing through application. Adv Funct Mater. 2020;30(32):2000639. 32802013 10.1002/adfm.202000639 PMC7418561

[52] Bernal PN, Delrot P, Loterie D, Li Y, Malda J, Moser C, Levato R. Volumetric bioprinting of complex living-tissue constructs within seconds. Adv Mater. 2019;31(42):1904209. 10.1002/adma.201904209 31423698

[53] Ma J, Huang C. Composition and mechanism of three-dimensional hydrogel system in regulating stem cell fate. Tissue Eng Part B Rev. 2020;26(6):498–518. 32272868 10.1089/ten.TEB.2020.0021

[54] Huang D, Li Y, Ma Z, Lin H, Zhu X, Xiao Y, Zhang X. Collagen hydrogel viscoelasticity regulates MSC chondrogenesis in a ROCK-dependent manner. Sci Adv. 2023;9(6): Article eade9497. 36763657 10.1126/sciadv.ade9497 PMC9916999

[55] Arango Duque G, Descoteaux A. Macrophage cytokines: Involvement in immunity and infectious diseases. Front Immunol. 2014;5:491. 25339958 10.3389/fimmu.2014.00491 PMC4188125

[56] Goldring MB, Otero M. Inflammation in osteoarthritis. Curr Opin Rheumatol. 2011;23(5):471–478. 21788902 10.1097/BOR.0b013e328349c2b1 PMC3937875

[57] Laverty S, Girard CA, Williams JM, Hunziker EB, Pritzker KP. The OARSI histopathology initiative—Recommendations for histological assessments of osteoarthritis in the rabbit. Osteoarthr Cartil. 2010;18:S53–S65. 10.1016/j.joca.2010.05.029 20864023

[58] Rebai MA, Sahnoun N, Abdelhedi O, Keskes K, Charfi S, Slimi F, Frikha R, Keskes H. Animal models of osteoarthritis: Characterization of a model induced by Mono-Iodo-Acetate injected in rabbits. Libyan J Med. 2020;15(1): Article 1753943. 32281500 10.1080/19932820.2020.1753943 PMC7178858

