# Supplementary material for: Programmable Macrophage Mimics for Inflammatory Meniscus Regeneration via Nanotherapy
Source: Research (Wash D C). 2026 Jan 12;9:1056. doi: 10.34133/research.1056 (PMC12794206; doi:10.34133/research.1056)
Supplement: Supplementary 1 — Figs. S1 to S10 [file research.1056.f1.docx]

SUPPORTING INFORMATION

**Programmable** **macrophage mimics for** **inflammatory meniscus regeneration via nano****therapy**

**Xujie Lu^1^**^†^**, Zheng Ci^1^**^†^**, Bohui Li^2^**^†^**, Yajie Wang^2^, Di Wang^2^, Xiang Zhang^1^, Yingying Huo^1*^, Xiansong Wang^1^, Guangdong Zhou^2,3*^, Yujie Hua^1*^**

^1^Department of Plastic and Reconstructive Surgery, Shanghai Ninth People’s Hospital, Shanghai Key Laboratory of Tissue Engineering, Shanghai Jiao Tong University School of Medicine, Shanghai 200001, PR China.

^2^Plastic Surgery Institute, Shandong Provincial Key Laboratory for Tissue Regeneration and Repair & Reconstruction (Under Preparation), Shandong Second Medical University, Weifang, Shandong 261053, PR China.

^3^National Tissue Engineering Center of China, Shanghai 200241, P. R. China.

* Corresponding author. E-mail address: [yujiehua@shsmu.edu.cn](mailto:yujiehua@shsmu.edu.cn)(Y.H)[guangdongzhou@126.com](mailto:guangdongzhou@126.com)(G.Z)[yingying_huo@shsmu.edu.cn](mailto:yingying_huo@shsmu.edu.cn)(Y.H).

^†^These authors contributed equally to this work.


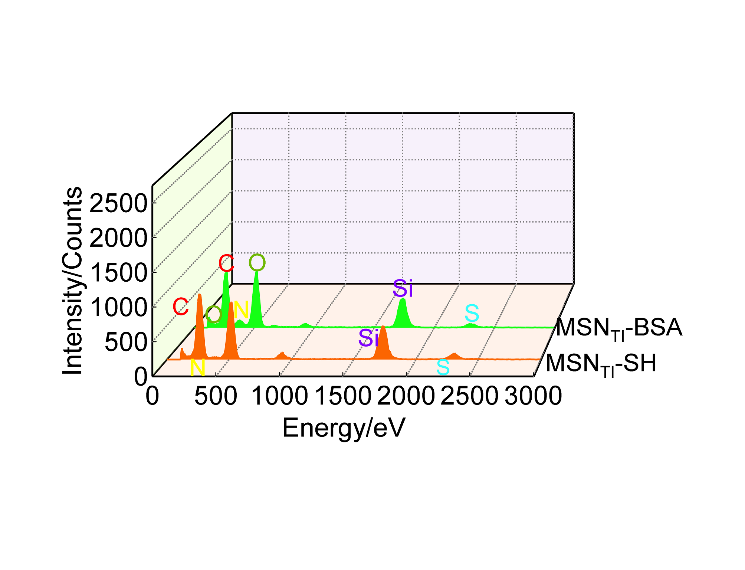


**Fig. S1.** Elemental percentages of EDS experiments of MSN_TI_-SH and MSN_TI_-BSA nanoparticles.


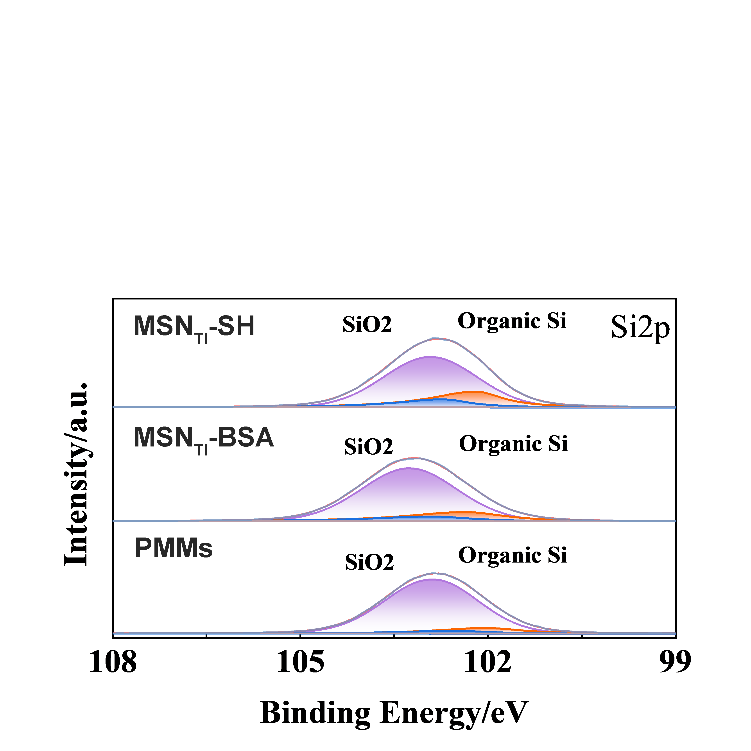


**Fig. S2.** XPS analysis of Si 2P in the different groups of MSN_TI_-SH, MSN_TI_-BSA, and MSN_TI_-BPEI nanoparticles.


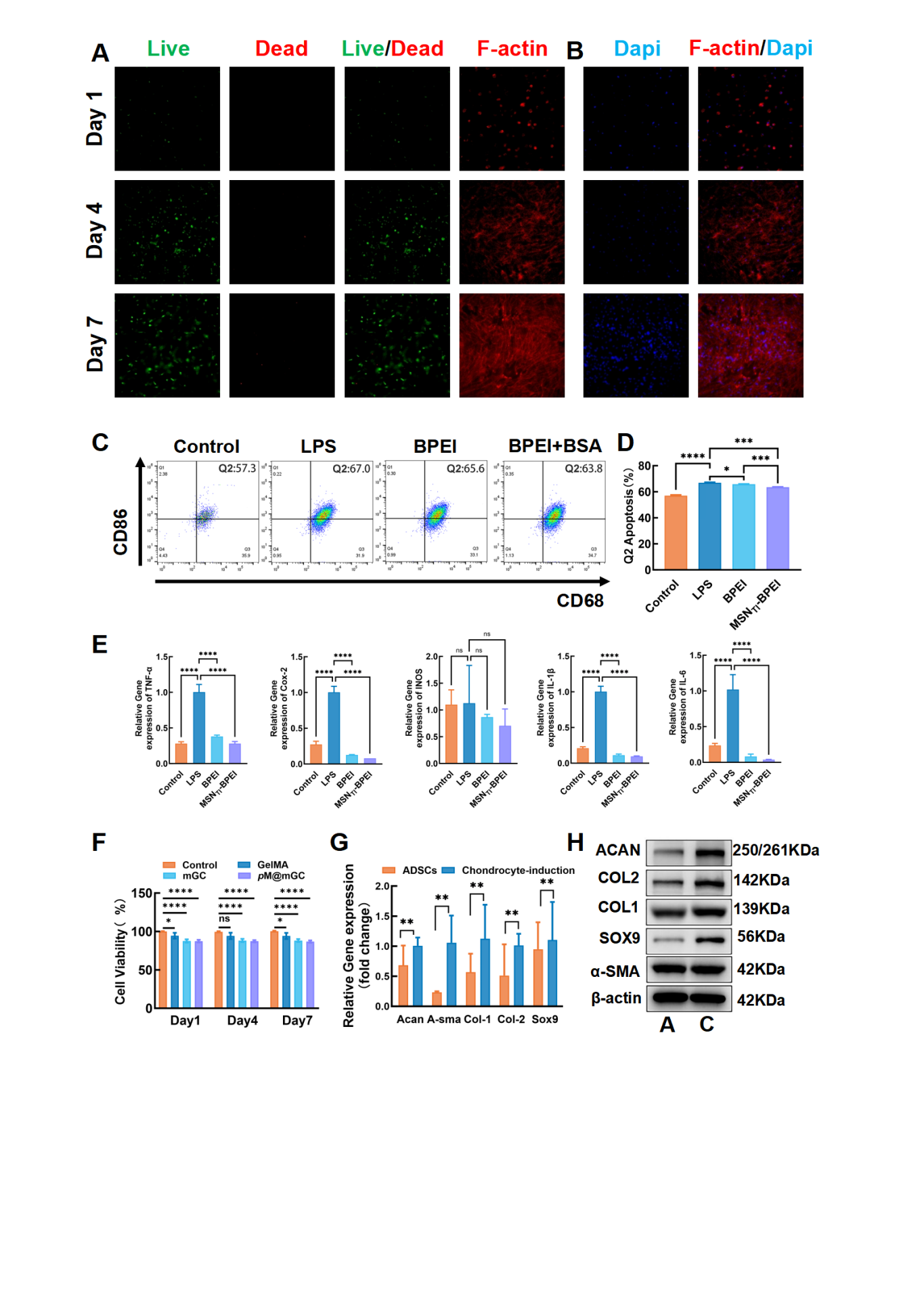


**Fig. S3.** Live/dead cell staining and F-actin staining of ADSCs co-cultured with PMMs@mGC hydrogel extracts for 1, 4, and 7 days.


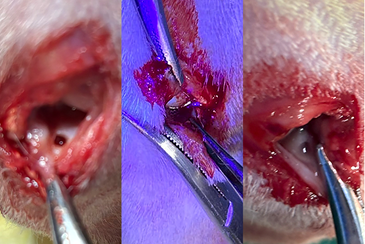


**Fig. S4.** Intraoperative flow chart of meniscus defect repair using PMMs@mGC hydrogels.


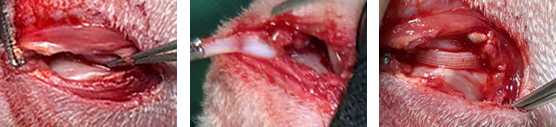


**Fig. S5.** Intraoperative flow chart of total meniscus replacement using ADSCs-loaded PMMs@mGC-PCL scaffolds.


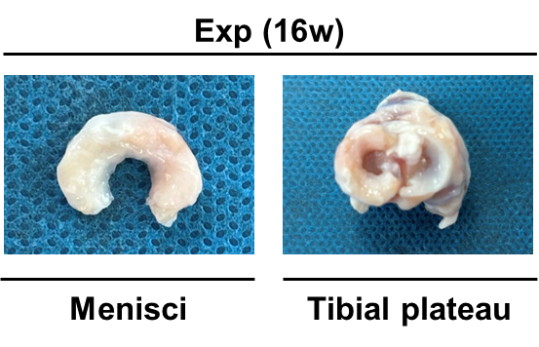


**Fig. S6.** Representative gross view of regenerative meniscus after 16-weeks post-surgery in Exp group at week 16.


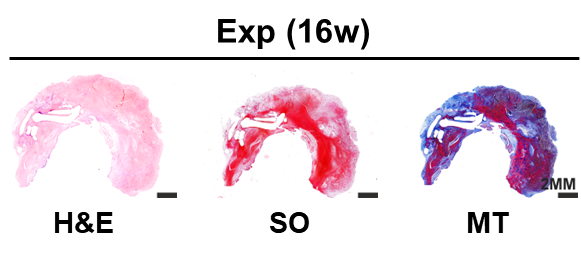


**Fig. S7.** Histological staining of the meniscus cartilage repair evaluated by hematoxylin and eosin (H&E), Safranin-O (SO), and Masson's trichrome (MT) staining at week 16.


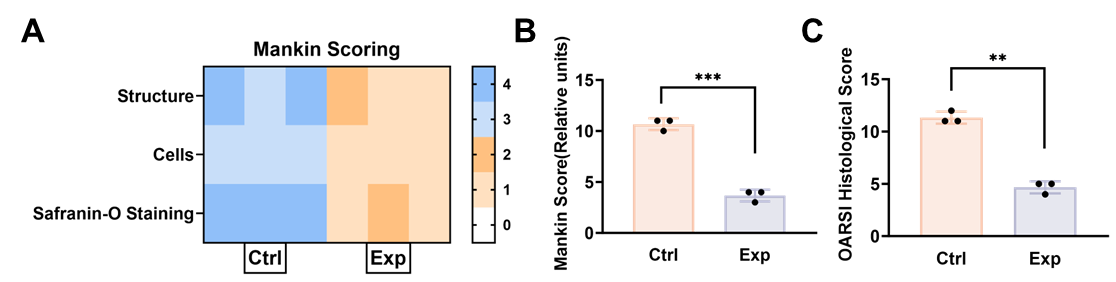


**Fig. S8.** Heatmap (A) and histogram (B) of Mankin scores for each group; C) histogram of OARSI scores for each group. **P < 0.01, ***P < 0.001, and ****P < 0.0001, ns = no significance.


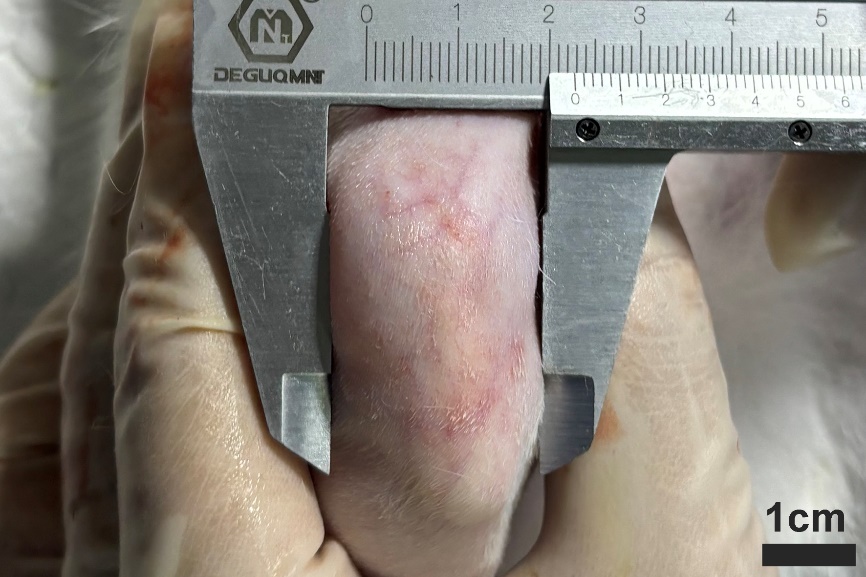


**Fig. S9.** Two weeks after the injection of type II collagenase, the swelling situation of the Exp group.


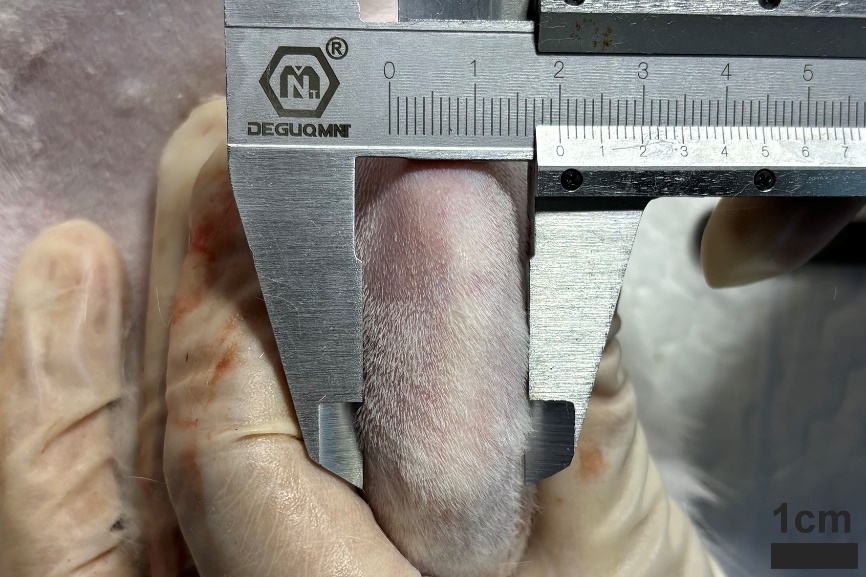


**Fig. S10.** The swelling situation of the Control group.
